# Differences in the neural basis and transcriptomic patterns in acute and persistent pain-related anxiety-like behaviors

**DOI:** 10.3389/fnmol.2023.1185243

**Published:** 2023-05-26

**Authors:** Shunchang Fang, Yuxin Qin, Shana Yang, Hongyang Zhang, Jieyan Zheng, Songhai Wen, Weimin Li, Zirui Liang, Xiaomin Zhang, Boxing Li, Lianyan Huang

**Affiliations:** ^1^Guangdong Provincial Key Laboratory of Brain Function and Disease, Neuroscience Program, Zhongshan School of Medicine and the Fifth Affiliated Hospital, Sun Yat-sen University, Guangzhou, China; ^2^Medical College, Jiaying University, Meizhou, China; ^3^Advanced Medical Technology Center, The First Affiliated Hospital, Zhongshan School of Medicine, Sun Yat-sen University, Guangzhou, China

**Keywords:** anxiety-like behavior, acute pain, persistent pain, BNST, mPFC

## Abstract

**Background:**

Both acute and persistent pain is associated with anxiety in clinical observations, but whether the underlying neural mechanisms differ is poorly understood.

**Methods:**

We used formalin or complete Freund’s adjuvant (CFA) to induce acute or persistent pain. Behavioral performance was assessed by the paw withdrawal threshold (PWT), open field (OF), and elevated plus maze (EPM) tests. C-Fos staining was used to identify the activated brain regions. Chemogenetic inhibition was further performed to examine the necessity of brain regions in behaviors. RNA sequencing (RNA-seq) was used to identify the transcriptomic changes.

**Results:**

Both acute and persistent pain could lead to anxiety-like behavior in mice. The c-Fos expression indicates that the bed nucleus of the stria terminalis (BNST) is activated only in acute pain, whereas the medial prefrontal cortex (mPFC) is activated only in persistent pain. Chemogenetic manipulation reveals that the activation of the BNST excitatory neurons is required for acute pain-induced anxiety-like behaviors. In contrast, the activation of the prelimbic mPFC excitatory neurons is essential for persistent pain-induced anxiety-like behaviors. RNA-seq reveals that acute and persistent pain induces differential gene expression changes and protein–protein interaction networks in the BNST and prelimbic mPFC. The genes relevant to neuronal functions might underline the differential activation of the BNST and prelimbic mPFC in different pain models, and be involved in acute and persistent pain-related anxiety-like behaviors.

**Conclusion:**

Distinct brain regions and gene expression patterns are involved in acute and persistent pain-related anxiety-like behaviors.

## 1. Introduction

Pain is an unpleasant multidimensional experience that comprises sensory and affective components (Talbot et al., 2019). Accumulating evidence indicates that pain and emotional disorders are highly comorbid. For example, pain is comorbid with anxiety or depression in over 50% of patients (Roy-Byrne et al., 2008; Asmundson and Katz, 2009). The interaction with negative emotions exacerbates pain sensation and results in poor physical and functional outcomes, seriously reducing patients’ quality of life (Becker et al., 2018; Michaelides and Zis, 2019; Notaro et al., 2021). While much is known about the mechanisms underlying the transmission of pain sensation (D'mello and Dickenson, 2008), the neural underpinnings of pain-related negative emotions, such as anxiety, are largely unknown.

Anxiety-like behaviors induced by persistent and acute pain have received extensive clinical attention. For example, persistent pain caused by injuries or diseases, including lower back pain, postherpetic neuralgia, and fibromyalgia, has been associated with anxiety-like behaviors (Hauser et al., 2015; Bunzli et al., 2017; Kohrt et al., 2018; Derry et al., 2019; Kaur et al., 2019; Cohen et al., 2021). In addition, previous studies indicate that acute pain caused by trauma, acute medical conditions, or surgeries (Carr et al., 2005; Eisenach et al., 2008; Hall et al., 2011; Luo et al., 2015; Pope et al., 2017) plays a pivotal role in emotional disorders (Lorentzen et al., 2012; Gan et al., 2014; Luo et al., 2015; Michaelides and Zis, 2019). However, although both persistent and acute pain induces anxiety-like behaviors, the differences in the neural mechanisms underlying these two types of pain-induced anxiety-like behaviors are poorly understood.

It is well-known that pain processing and its related emotional experience are controlled by the activation of the brain network (Tracey, 2008; Seifert and Maihofner, 2009; Flodin et al., 2016; Peyron and Fauchon, 2019). However, the brain activity patterns and regions underlying the affective dimension of pain remain controversial. Functional imaging studies showed that the prefrontal cortex (PFC), anterior cingulate cortex (ACC), posterior parts of the insula, and limbic brain played key roles in the emotional regulation of pain (Melzack, 2001; Apkarian et al., 2005; Lee et al., 2015; Flodin et al., 2016). However, humans subjected to acute thermal pain did not show the activation of the key nodes in the limbic brain (Apkarian et al., 2005) (Wager et al., 2013). Therefore, it remains unclear whether different types of pain share similar brain regions in regulating anxiety-like behaviors, or whether they employ different neural mechanisms.

In this study, we constructed the acute and persistent pain models by formalin or CFA injections, respectively, and compared the neural basis of anxiety-like behaviors induced by the two models. Our results showed that the BNST mainly participates in acute pain-induced anxiety-like behaviors, with no effect on persistent pain-induced anxiety-like behaviors. In contrast, the prelimbic mPFC controlled persistent pain-induced anxiety-like behaviors. Moreover, the two pain models elicit distinct transcriptomic changes in the BNST and prelimbic mPFC. These findings reveal that acute and persistent pain engage different neural mechanisms in inducing anxiety-like behaviors, and provide potential therapeutic strategies for pain-related anxiety-like behaviors.

## 2. Materials and methods

### 2.1. Animals

All animal experiments were approved by the Institutional Animal Care and Use Committee of Sun Yat-sen University. Adult male C57BL/6 mice (6–8 weeks) from the Laboratory Animal Center of Sun Yat-sen University (Guangzhou, China) were used in this study. All mice were housed in groups of 5–6 per cage on a 12-h light/dark cycle (temperature, 22–24°C; humidity, 40–60%), having access to food and water available *ad libitum*. Mice in the persistent pain model received CFA injection (10 μL, 50% in saline) into the plantar surface of the hind paw (Wang et al., 2015). Mice in the acute pain model received formalin injection (10 μL, 1% in saline) into the plantar surface of the hind paw (Luo et al., 2015). The control group referred to mice without CFA or formalin injection. To minimize the impact of nociceptive behavioral tests on subsequent anxiety-like behavioral tests, two groups of mice were used: one underwent the von Frey test, and the other underwent the anxiety-related behavioral tests.

### 2.2. Avertin preparation and use

Avertin was prepared following the method described in previous studies (Papaioannou and Fox, 1993; Lieggi et al., 2005a,b). Briefly, 2.5 grams of 2,2,2-tribromoethanol (Sigma T48402, Lot#MKBZ3419V) was dissolved in 5 mL of tert-amyl alcohol (Sigma 240,486, Lot#STBJ9487) at room temperature. The solution was vigorously stirred in a dark bottle, and sterile PBS was continuously added while stirring to reach a final volume of 200 mL. The resulting solution was filter sterilized through a 0.2 μm filter and aliquoted into sterile, light-protected containers. In mice, the dosage administered was 250 mg/kg given intraperitoneally (IP).

### 2.3. Nociceptive behavioral testing

Paw withdrawal threshold (PWT) was tested using von Frey filaments (ranging from 0.04 to 2 g) with motified procedures following the Simplified Up-Down method (SUDO) described previously (Bonin et al., 2014). Briefly, the mice were allowed to habituate to the environment for 30 min before test. For each stimulation, von Frey filaments were applied to the plantar surface of the hind paw for up to 5 s, or until paw withdrawal. If a withdrawal occurred, a lighter filament was used for subsequent stimulations in the same paw and condition, whereas a higher force filament was used if no withdrawal was observed. The paw withdrawal threshold was evaluated using the last filament value +/−0.5 depending on the value of the fifth filament.

### 2.4. Open-field test (OF)

The open field test is conducted in a chamber (35 cm × 35 cm × 23 cm) in a quiet experimental room. During the test period, each mouse was placed in the center area of the chamber, and the behavior of the mouse was recorded for 10 min using a camera fixed above the chamber. The behavior was analyzed by TopScan software (CleverSys Inc., VA, United States). Analyzed parameters included the total distance in the chamber, the distance across, and the time spent in the center area (50% of the total area). After each test, the chamber was cleaned using 70% ethanol.

### 2.5. Elevated plus maze test (EPM)

The elevated plus maze contained four arms, including two opposing closed arms (30 cm × 5 cm) and two opposing closed arms (30 cm × 5 cm). The two closed arms were surrounded by opaque walls (height 16 cm). A central zone (5 cm × 5 cm) is an area communicated by the four arms. The apparatus was located 50 cm above the ground. During the testing period, each mouse was allowed to move freely in all arms for 5 min. The exploratory behavior of each mouse was recorded using a camera fixed above the apparatus. Analyzed parameters included the time spent in open arms and the number of entries in the open arms. After each test, the EPM was cleaned using 70% ethanol.

### 2.6. Behavioral recording and analyzing

The behaviors of each mouse were recorded using SuperMaze video software (XinSoft Inc., Shanghai, China). After that, all the videos were analyzed using TopScan software (CleverSys Inc., VA, United States). The behavioral tests and analyses were performed by experimenters blinded to the experimental groups.

### 2.7. Stereotaxic surgery and viral injection

Mice were anesthetized with Avertin (250 mg/kg, i.p.) and placed in a stereotactic setup (Nanjing ThinkerTech, China). After stereotaxic location and craniotomy hole, a micropipette (WPI, Sarasota, FL) was used to inject a virus into target brain regions. For suppressing excitatory neurons in the mPFC or the BNST, mice were bilaterally injected in the prelimbic mPFC (2.15 mm anterior, 0.2 mm lateral and 1.7 mm in depth) or BNST (0.10 mm anterior, 0.75 mm lateral and 4.15 mm in depth) with 0.2 μL of AAV2/9-CaMKII-hM4d (Gi)-mCherry (titers: 2–5 × 10^12^ particles/ml) virus (BrainVTA, China). For the control experiment, mice were bilaterally injected with 0.2 μL of AAV2/9-CaMKII-mCherry (titers: 2–5 × 10^12^ particles/ml) virus (BrainVTA, China). After that, each mouse was recovered for 21 days.

### 2.8. Chemogenetic manipulation

The designer drug Clozapine-N-Oxide (CNO, 3 mg/kg, i.p.; Tocris Bioscience) was injected 40 min before OF or EPM tests.

### 2.9. Immunohistochemistry

The mice brains were collected in Avertin anesthesia and euthanasia methods. Mice received Avertin injection for 5 min and were transcardially perfused with phosphate-buffered saline when fully sedated, as measured by a lack of active paw reflex. The brains were fixed with 4% paraformaldehyde in PBS (PFA) at 4°C overnight. Afterward, brains were transferred into 30% sucrose in PBS solution at 4°C for 2–3 days. Whole brain regions were cut into 45 μm thick coronal sections on a freezing microtome (Leica, CM1950). For immunostaining, the slices were washed in PBS, and then incubated in a blocking solution (5% donkey serum in 0.1% Triton X-100) containing primary antibody at 4°C overnight. Following washing with PBS, the slices were incubated in secondary antibody for 2 h as well as stained with DAPI (1, 10,000, Invitrogen, United States) for 5 min at room temperature. The primary antibody was a rabbit anti-c-Fos (1, 500, Cell Signaling Technology, United States), and the secondary was donkey anti-rabbit coupled to Alexa 647 (1, 500, Invitrogen, United States). After mounting onto glass slides, the brain slices were examined using an ECLIPSE Ni-E (Nikon, Japan) microscope. For injection site verification, the brain slices were imaged on AxioScan.Z1 (Zeiss, United States).

The number of c-Fos-positive cells per section per area (mm^2^) was counted for each analyzed area, and the mean number was compared between control and pain conditions. A total of 9 brain slices from 3 animals per condition were quantified using the NIH ImageJ software.

### 2.10. Tissue dissection and RNA extraction and sequencing

Mice in the persistent pain group were sacrificed 72 h after CFA exposure. Mice in the acute pain group were sacrificed 24 h after formalin injection. Mice without CFA or formalin injection were sacrificed as the control group. Three brains of each group were cut on a brain matrix (RWD, China) in ice-cold 0.9% saline. The prelimbic mPFC and BNST of each brain were dissected and kept on TRIzol at −80°C. The samples were submitted to Berry Genomics Co., Ltd. (Beijing, China) for RNA extraction following the manufacturer’s instructions. mRNA library was prepared by the VAHTST mRNA-seq V3 Library Prep Kit for Illumina according to the manufacturer’s instructions. The length of the reads is 150 bp. The supplemental file (All_samples_featureCounts_counts.txt) contains the number of reads obtained from each sample. The raw data and the alignment detail can be accessed via GEO accession GSE227946.

### 2.11. RNA-sequencing data analysis

Paired-end raw reads were first trimmed using Trimmomatic v0.39 (Bolger et al., 2014) to remove the adapter sequence and aligned to the GRCm38 mouse reference genome by HISAT2 v2.2.1 (Kim et al., 2019). Samtools v1.9 (Li et al., 2009) and featureCounts v2.0.1 (Liao et al., 2014) were used to sort and obtain the primary count table. Differential gene expression analysis was conducted on R by DESeq2 v1.34 (Love et al., 2014). Genes with adjusted value of p (Benjamin–Hochberg corrected) less than 0.05 and absolute log2FC greater than 0.5 were considered differentially expressed genes. GSEA was performed by the gseGO function in clusterProfiler v4.2.2 (Yu et al., 2012) using org.Mm.eg. db v3.14.0 as a gene ontology data resource. Enriched GO terms with adjusted value of p less than 0.2 were considered to be significant in the results. The top 300 differential expressed genes sorted by log2 fold change (log2FC) with adjusted value of ps less than 0.05 were used for further protein interaction network analysis conducted by StringApp v2.0.1 in Cytoscape v3.9.1 (Szklarczyk et al., 2019). Single nodes and small networks without connection to the major network were neglected.

### 2.12. Statistical analysis

The statistical data were calculated using GraphPad Prism (Version 7.0). Data collection and analyses were performed blinded to the conditions of the experiments. The data are expressed as means ± SEM. For comparing the experimental data between two groups, the two-sided Student’s t-test was used. The criterion for statistically significant was *p*-value <0.05. These representative genes comparing the CFA and Formalin treatment are all with adjusted value of *p* <0.05 and log2FC > 0.5 according to the calculation of DESeq2.

## 3. Results

### 3.1. Both acute pain and persistent pain induce anxiety-like behaviors

**Figure 1:**
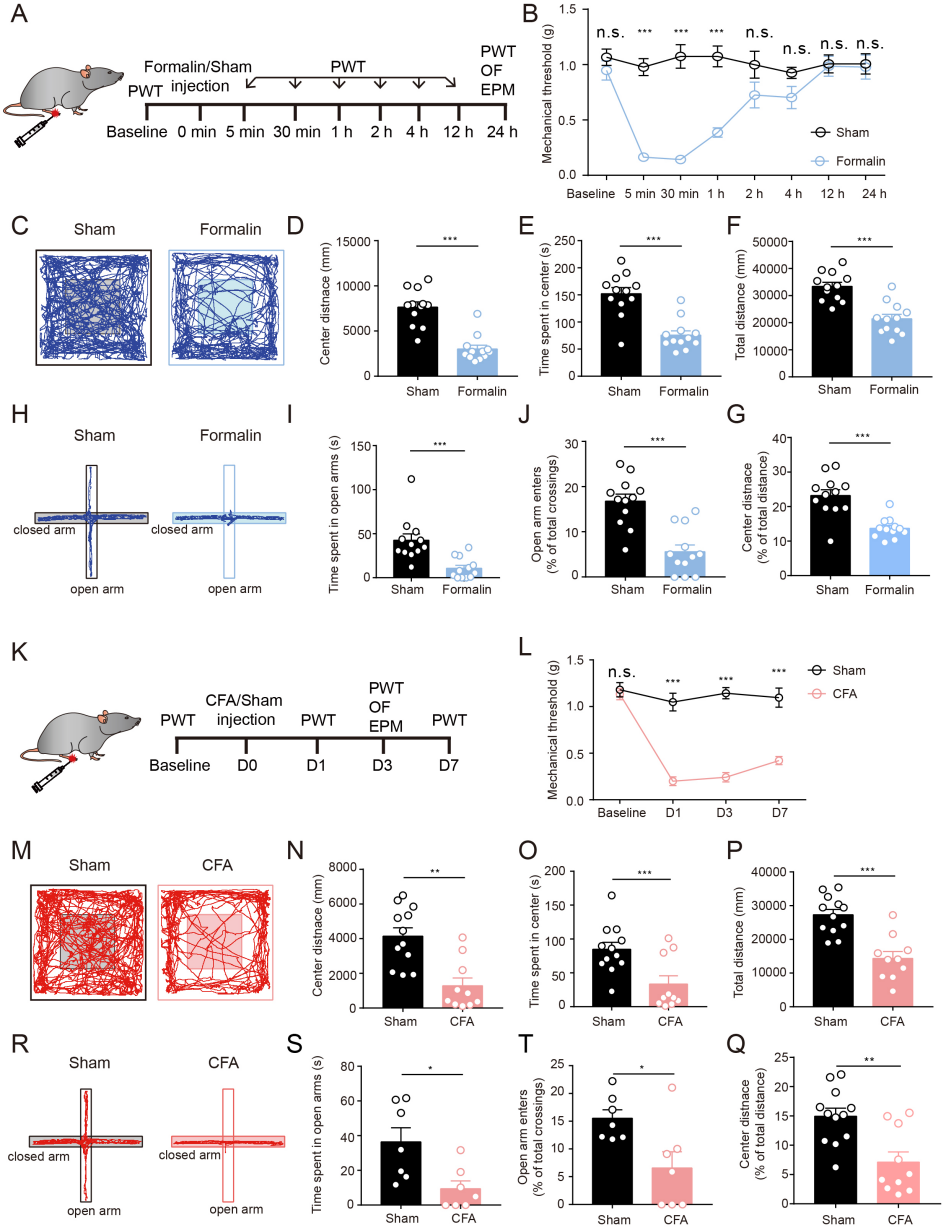
Both Acute Pain and Persistent Pain Induce Anxiety-Like Behaviors. **(A)** The timeline of formalin injection and behavioral tests. **(B)** Mechanical thresholds (withdrawal thresholds to mechanical stimulation) at different time points comparing formalin-injected and sham groups (sham group, *n* = 9; formalin group, *n* = 9). **(C)** Representative locomotion traces of sham and formalin-injected mice in the OF test. **(D–G)** The center distance **(D)**, center duration **(E)**, total distance **(F)**, and the percentage of center distance **(G)** in the OF test (sham group, *n* = 12; formalin group, *n* = 12). **(H)** Representative locomotion traces of sham and formalin-injected mice in the EPM test. **(I,J)** The time spent in open arms **(I)** and the percentage of open arm entries **(J)** in the EPM test (sham group, *n* = 12; formalin group, *n* = 12). **(K)** The timeline of CFA injection and behavior tests. **(L)** Mechanical thresholds at different time points, comparing CFA-injected and sham groups (sham group, n = 7; CFA group, n = 7). **(M)** Representative locomotion traces of sham and CFA-injected mice in the OF test. **(N-Q)** The center distance **(N)**, center duration **(O)**, total distance **(P)**, and the percentage of center distance **(Q)** in the OF test (sham group, *n* = 12; CFA group, *n* = 10). **(R)** Representative locomotion traces of sham and CFA-injected mice in the EPM test. **(S,T)** The time spent in open arms **(S)**, and the percentage of open arm entries **(T)** in the EPM test (sham group, *n* = 7; CFA group, *n* = 7). The data are expressed as means ± SEM. For comparing the experimental data between two groups, the two-sided Student’s t-test was used. Significance is shown as ^*^p < 0.05, ^**^p < 0.01, and ^***^p < 0.001.

To investigate whether acute and persistent pain could lead to anxiety-like behaviors in mice, we injected formalin or CFA into the paw of adult mice to induce acute and persistent pain, respectively (Luo et al., 2015; Wang et al., 2015). The paw withdrawal threshold (PWT) tests were performed to measure mechanical allodynia at different time points (Figure 1A). Formalin injection induced acute mechanical allodynia, which appeared 5 min after injection and lasted for about 2 h (Figure 1B). Mice exposed to CFA injections exhibited persistent pain and reduced mechanical thresholds in the PWT test for at least 7 days post-injection (Figures 1K,L). These results confirm the effects and duration of formalin and CFA in acute and persistent pain models.

To further investigate the acute and persistent pain-related anxiety-like behaviors, we performed 10 min of open field (OF) and 5 min of elevated plus maze (EPM) tests after acute or persistent pain was successfully induced (Figures 1A,K). We first examined the anxiety-like behaviors after acute pain. We found that 1-day of formalin injection, the mice showed a significant reduction in the center distance, center exploration time, and total distance in the OF test, suggesting that formalin-treated mice exhibited apparent anxiety-like behaviors after acute pain (Figures 1C–G). This was further supported by the results of EPM tests (Figures 1H–J). Compared to sham-treated mice, formalin-treated mice showed much fewer entries to the open arms and spent a much shorter duration in the open arms in the EPM tests (Figures 1H–J). These results indicate that acute pain leads to apparent anxiety-like behaviors in mice.

We further examined anxiety-like behaviors after persistent pain. We found that 3 days after CFA injection, the mice exhibited significantly lower total distance, center distance, and center exploration time in the OF tests (Figures 1M–Q). In addition, CFA-treated mice exhibited less exploration of the open arms and reduced entries to the open arms in the EPM tests (Figures 1R–T). These results indicate that persistent pain could also lead to apparent anxiety-like behaviors in mice.

Taken together, these results indicate that formalin and CFA injection could lead to acute and persistent pain, respectively, and that both formalin-induced acute pain and CFA-induced persistent pain result in anxiety-like behaviors in mice.

### 3.2. Acute and persistent pain activates distinct brain regions

**Figure 2:**
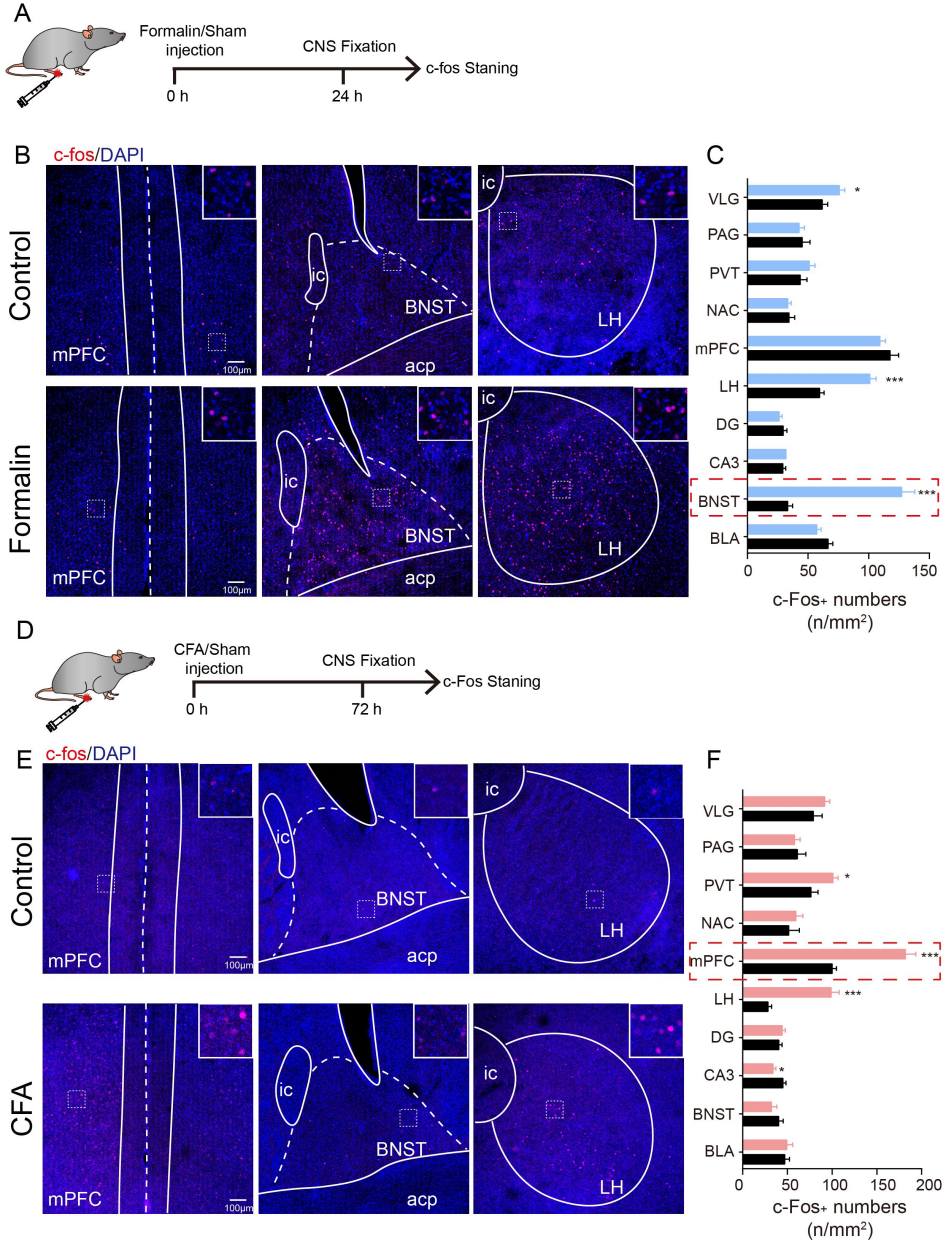
Acute and Persistent Pain Activates Distinct Brain Regions. **(A)** The timeline of formalin injection and c-Fos staining. **(B)** Representative images of c-Fos staining in the mPFC, BNST, and LH after saline and formalin injection. Insert, magnification of the squared region, indicating the co-localization of c-Fos and DAPI. **(C)** Quantification of c-Fos expression in the VLG, PAG, PVT, NAC, mPFC, LH, DG, CA3, BNST, and BLA after saline and CFA injection (*N* = 3, *n* = 9 slices each group). Scale bar = 100 μm. **(D)** The timeline of CFA injection and c-Fos staining. **(E)** Representative images of c-Fos staining in the mPFC, BNST, and LH after saline and CFA injection. Insert, magnification of the squared region, indicating the co-localization of c-Fos and DAPI. **(F)** Quantification of c-Fos expression in the VLG, PAG, PVT, NAC, mPFC, LH, DG, CA3, BNST, and BLA after saline and CFA injection (*N* = 3, *n* = 9 slices each group). Scale bar = 100 μm. The data are expressed as means ± SEM. For comparing the experimental data between two groups, the two-sided Student’s t-test was used. Significance is shown as ^*^p < 0.05, ^**^ *p* < 0.01, and ^***^ *p* < 0.001.

To identify the brain regions potentially contributing to anxiety-like behaviors in the acute and persistent pain models, we performed c-Fos staining to probe the activated brain regions (Figures 2A,D). Strikingly, acute and persistent pain activated distinct brain regions. In the formalin-induced acute pain group, higher c-Fos expression was observed in the BNST, ventrolateral geniculate nucleus (VLG), and lateral hypothalamus (LH) (Figures 2B,C). Among these regions, the BNST exhibited the most significant increase in c-Fos expression (Figures 2B,C). On the other hand, in the CFA-induced persistent pain group, c-Fos expression was higher in the paraventricular thalamus (PVT), mPFC, and LH (Figures 2E,F). Among them, the mPFC showed the most significant increase in c-Foc expression (Figures 2E,F). These results indicate that acute and persistent pain activate distinct brain regions in the brain, with the most significant activation in the BNST in acute pain, and the mPFC in persistent pain.

### 3.3. The neuronal activity of excitatory neurons in the BNST is required for acute pain-induced anxiety-like behaviors

**Figure 3:**
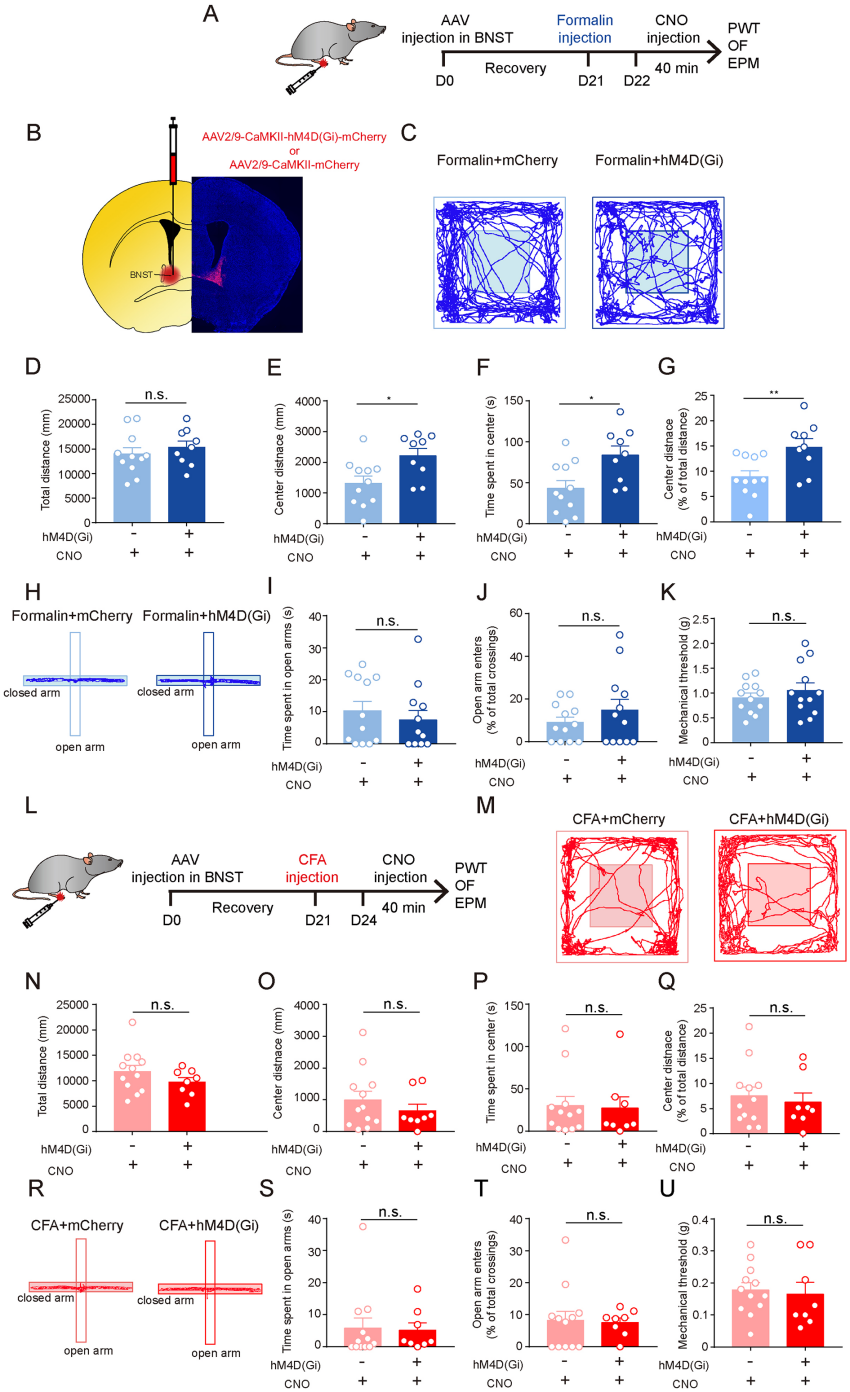
The Neuronal Activity of Excitatory Neurons in the BNST is Required for Acute Pain-Induced Anxiety-Like Behaviors. **(A)** The timeline of virus, formalin and CNO injection, and behavioral tests. **(B)** Diagram and a representative image showing injection of AAV-CaMKII-hM4d(Gi)-mCherry or (Continued)FIGURE 3 (Continued)AAV-CaMKII-mCherry into the BNST. Scale bar = 500 μm. **(C)** Representative locomotion traces of mCherry- (light) and hM4d(Gi)-expressing (dark) mice in the OF test. **(D–G)** The total distance **(D)**, center distance **(E)**, center duration **(F)** and the percentage of center distance **(G)** of mCherry- and hM4d(Gi)-expressing mice in the OF test (sham group, *n* = 11; formalin group, *n* = 9). **(H)** Representative locomotion traces of mCherry- (light) and hM4d(Gi)-expressing (dark) mice in the EPM test. **(I,J)** The time spent in open arms **(I)**, and the percentage of open-arm entries **(J)** of mCherry- and hM4d(Gi)-expressing mice in the EPM test (sham group, *n* = 12; formalin group, *n* = 12). **(K)** Pain threshold for mechanical allodynia of mCherry- and hM4d(Gi)-expressing mice in acute pain model (sham group, *n* = 12; formalin group, *n* = 12). **(L)** The timeline of virus, CFA and CNO injection, and behavioral tests. **(M)** Representative locomotion traces of mCherry- (light) and hM4d(Gi)-expressing (dark) mice in the OF test. **(N–Q)** The total distance **(N)**, center distance **(O)**, center duration **(P)**, and and the percentage of center distance **(Q)** of mCherry- and hM4d(Gi)-expressing mice in the OF test (sham group, *n* = 12; CFA group, *n* = 8). **(R)** Representative locomotion traces of mCherry- (light) and hM4d(Gi)-expressing (dark) mice in the EPM test. **(S,T)** The time spent in open arms **(S)** and the percentage of open-arm entries **(T)** of mCherry- and hM4d(Gi)-expressing mice in the EPM test (sham group, *n* = 12; CFA group, *n* = 8). **(U)** Pain threshold for mechanical allodynia of mCherry- and hM4d(Gi)-expressing mice in persistent pain model (sham group, *n* = 12; CFA group, *n* = 8). The data are expressed as means ± SEM. For comparing the experimental data between two groups, the two-sided Student’s t-test was used. Significance is shown as ^*^p < 0.05 and ^**^ *p* < 0.01.

Given that the activation of excitatory neurons in the BNST (BNST^EXT^ neurons) promotes anxiety-like behaviors (Kim et al., 2013; Ch'ng et al., 2018; Kim and Kim, 2021), and that the increase in c-Fos expression was the most significant in the BNST after formalin injection (Figures 2B,C), we hypothesized that the activation of BNST^EXT^ neurons is responsible for acute pain-induced anxiety-like behaviors. To test this, we expressed the inhibitory Designer Receptors Exclusively Activated by Designer Drugs receptor (hM4Di DREADD) in the BNST^EXT^ neurons. Three weeks later, the mice were injected with formalin to induce acute pain (Figures 3A,B). We found that, after Clozapine-N-Oxide (CNO) injection, hM4Di-mediated inhibition of BNST^EXT^ neurons did not change pain sensitivity in the PWT test (Figure 3K), but significantly increased center exploration and center duration in the OF test (Figures 3C,E–G), without affecting the total distance in the OF test or the performance in the EPM test (Figures 3D,H–J). These results suggest that the activation of the BNST^EXT^ neurons is required for acute pain-induced anxiety-like behaviors.

We further investigated the role of the BNST^EXT^ neurons in persistent pain-induced anxiety-like behaviors (Figure 3L). Strikingly, compared to mCherry-expressing controls, hM4Di-mediated inhibition of BNST^EXT^ neurons did not change the performance in OF, EPM, and PWT tests of CFA-treated mice (Figures 3M–U). These results were consistent with the findings that c-Fos expression was not altered in the BNST^EXT^ neurons in CFA-induced persistent pain (Figure 2F).

Taken together, these results indicate that the neuronal activity of the BNST^EXT^ neurons is responsible for anxiety-like behaviors induced by acute pain but not by persistent pain.

### 3.4. The neuronal activity of excitatory neurons in the prelimbic mPFC is required for persistent pain-induced anxiety-like behaviors

**Figure 4:**
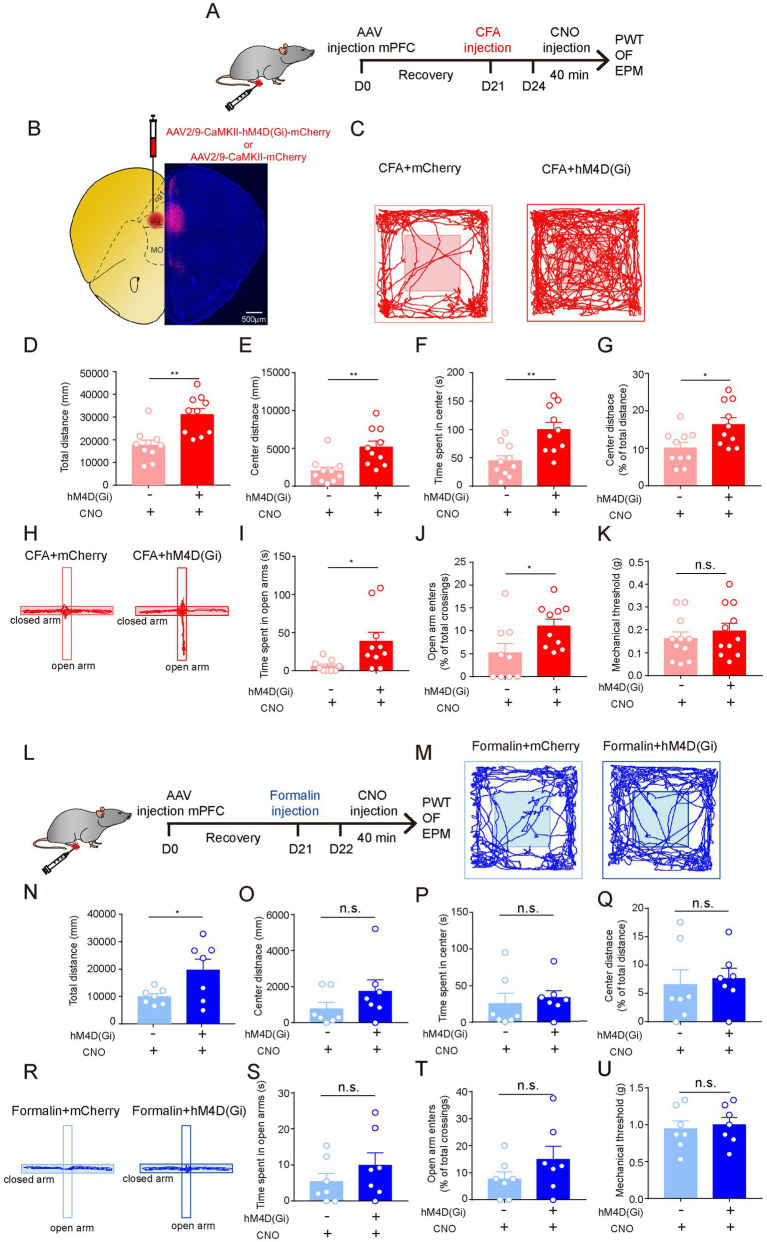
The Neuronal Activity of Excitatory Neurons in the Prelimbic mPFC is Required for Persistent Pain-Induced Anxiety-Like Behaviors. **(A)** The timeline of virus, CFA and CNO injection, and behaviour tests. **(B)** Diagram and a representative image showing injection of AAV-CaMKII-hM4d(Gi)-mCherry or (Continued)FIGURE 4 (Continued)AAV-CaMKII-mCherry into the prelimbic mPFC. Scale bar = 500 μm. **(C)** Representative locomotion traces of mCherry- (light) and hM4d(Gi)-expressing (dark) mice in the OF test. **(D–G)** The total distance **(D)**, center distance **(E)**, center duration **(F)**, and the percentage of center distance **(G)** of mCherry- and hM4d(Gi)-expressing mice in the OF test (sham group, *n* = 10; CFA group, *n* = 10). **(H)** Representative locomotion traces of mCherry- (light) and hM4d(Gi)-expressing (dark) mice in the EPM test. **(I,J)** The time spent in open arms **(I)** and the percentage of open-arm entries **(J)** of mCherry- and hM4d(Gi)-expressing mice in the EPM test (sham group, *n* = 9; CFA group, *n* = 10). **(K)** Pain threshold for mechanical allodynia of mCherry- and hM4d(Gi)-expressing mice in persistent pain model (sham group, *n* = 11; CFA group, *n* = 11). **(L)** The timeline of virus, formalin and CNO injection, and behavioral tests. **(M)** Representative locomotion traces of mCherry- (light) and hM4d(Gi)-expressing (dark) mice in the OF test. **(N-Q)** The total distance **(N)**, center distance **(O)** and center duration **(P)**, and the percentage of center distance **(Q)** of mCherry- and hM4d(Gi)-expressing mice in the OF test (sham group, *n* = 7; formalin group, *n* = 7). **(R)** Representative locomotion traces of mCherry- (light) and hM4d(Gi)-expressing (dark) mice in the EPM test. **(S,T)** The time spent in open arms **(S)** and the percentage of open-arm entries **(T)** of mCherry- and hM4d(Gi)-expressing mice in the EPM test (sham group, *n* = 7; formalin group, *n* = 7). **(U)** Pain threshold for mechanical allodynia of mCherry- and hM4d(Gi)-expressing mice in acute pain model (sham group, *n* = 7; formalin group, *n* = 7). The data are expressed as means ± SEM. For comparing the experimental data between two groups, the two-sided Student’s t-test was used. Significance is shown as ^*^p < 0.05 and ^**^ *p* < 0.01.

We further investigated the neural mechanisms underlying persistent pain-induced anxiety-like behaviors. Given that the increase in c-Fos expression was most significant in the mPFC after CFA injection (Figures 2E,F), and that the mPFC is a pivotal region for anxiety-like behaviors, we first examined whether silencing the excitatory neurons in the prelimbic cortex (mPFC^EXT^ neurons) could influence persistent pain-induced anxiety-like behaviors. We expressed hM4Di in the excitatory neurons in prelimbic cortex and treated the mice with CFA injection 3 weeks later (Figures 4A,B). Three days later, when persistent pain was obtained, CNO was injected into the mice, and behavioral tests were performed (Figure 4A). We found that hM4Di-mediated inhibition of mPFC^EXT^ neurons did not change pain sensitivity in the PWT test (Figure 4K) but significantly increased center exploration and center duration in the OF test (Figures 4C–G). Additionally, inhibition of excitatory neurons in prelimbic cortex led to significant increases in the open arm duration and entries in the EPM test (Figures 4H–J). These results suggest that the activation of the mPFC^EXT^ neurons is required for persistent pain-induced anxiety-like behaviors.

We further investigated the role of the mPFC^EXT^ neurons in acute pain-induced anxiety-like behaviors (Figure 4L). Strikingly, compared to mCherry-expressing controls, hM4Di-mediated inhibition of mPFC^EXT^ neurons did not change the performance in OF, EPM, and PWT tests of formalin-treated mice (Figures 4M–U). These results suggest that the excitatory neurons in prelimbic cortex are not involved in acute pain-induced anxiety-like behaviors.

Taken together, these results indicate that the neuronal activity of the mPFC^EXT^ neurons is responsible for anxiety-like behaviors induced by persistent pain but not by acute pain.

### 3.5. Acute and persistent pain elicits distinct transcriptomic changes in the BNST and prelimbic mPFC

**Figure 5:**
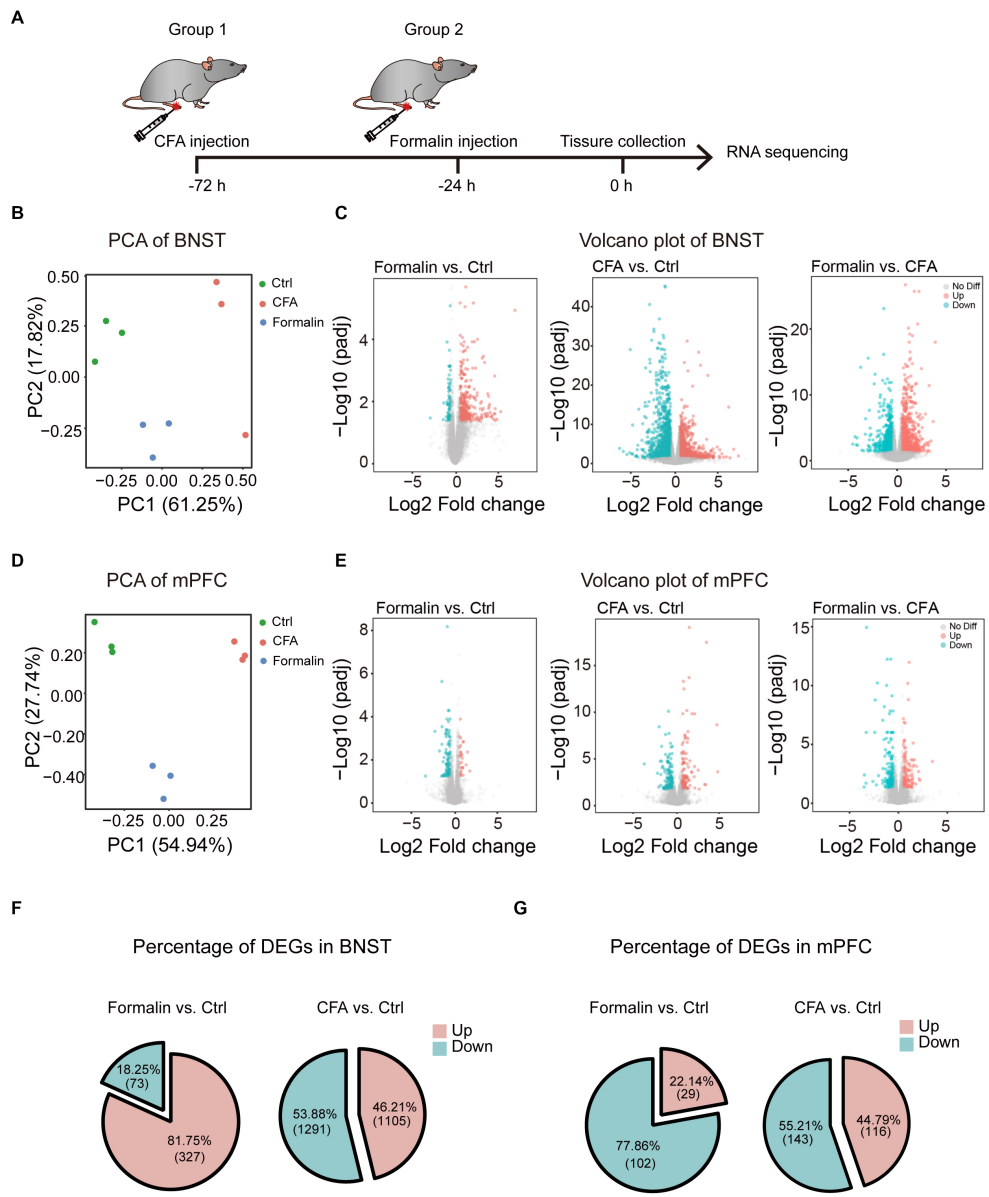
Distinct Transcriptomic Features of the BNST and Prelimbic mPFC in the Two Pain Models. **(A)** The timeline of formalin and CFA injection, tissue collection, and RNA sequencing (n = 3 mice each group). **(B)** PCA of gene expression in the BNST of formalin-, CFA-, and sham-treated mice. **(C)** Volcano plots comparing the DEGs in the BNST of formalin-, CFA-, and sham-treated mice. Blue dots and red dots represented the down-regulated and up-regulated genes. Grey dots represented genes not significantly changed. **(D)** PCA of gene expression in the prelimbic mPFC of formalin-, CFA-, and sham-treated mice. **(E)** Volcano plots comparing the DEGs in the prelimbic mPFC of formalin-, CFA-, and sham-treated mice. Blue dots and red dots represented the down-regulated and up-regulated genes. Grey dots represented genes not significantly changed. **(F)** The percentage of up-regulated (red) and down-regulated (blue) DEGs in the BNST. **(G)** The percentage of up-regulated (red) and down-regulated (blue) DEGs in the prelimbic mPFC.

The intriguing findings that the BNST and prelimbic mPFC are essential for acute and persistent pain-related anxiety-like behaviors, respectively, led us to investigate the underlying molecular mechanisms. The results that the BNST and prelimbic mPFC were activated differentially after acute and persistent pain raised the possibility that the transcriptomic patterns of these two brain regions may differ after acute or persistent pain induction. To test this hypothesis, we isolated the two regions from the brain and performed RNA sequencing (RNA-seq) to identify the transcriptomic differences after the mice were injected with formalin or CFA (Figure 5A). Principal component analysis (PCA) showed a clear separation among the acute pain, persistent pain, and control groups in the BNST (Figure 5B), indicating that acute and persistent pain induces different responses in the BNST. The volcano plot also showed that the gene expression patterns significantly differed between the CFA and formalin groups in the BNST (Figure 5C). Similarly, PCA results showed that acute and persistent pain also induced different responses in the prelimbic mPFC. The volcano plot also showed that the gene expression patterns significantly differed in the prelimbic mPFC after CFA and formalin treatment (Figures 5D,E). Interestingly, in the BNST, acute pain induced a larger proportion of upregulated genes than persistent pain (81.75% in the formalin model vs. 46.21% in the CFA model) (Figure 5F). On the contrary, a larger proportion of upregulated genes were observed in the prelimbic mPFC after persistent pain compared to acute pain (44.79% in the CFA model vs. 22.14% in the formalin model) (Figure 5G). These results indicated that acute and persistent pain induced differential gene expression patterns in the BNST and prelimbic mPFC, and that the differential activation of the BNST and prelimbic mPFC is associated with differential gene expression.

**Figure 6:**
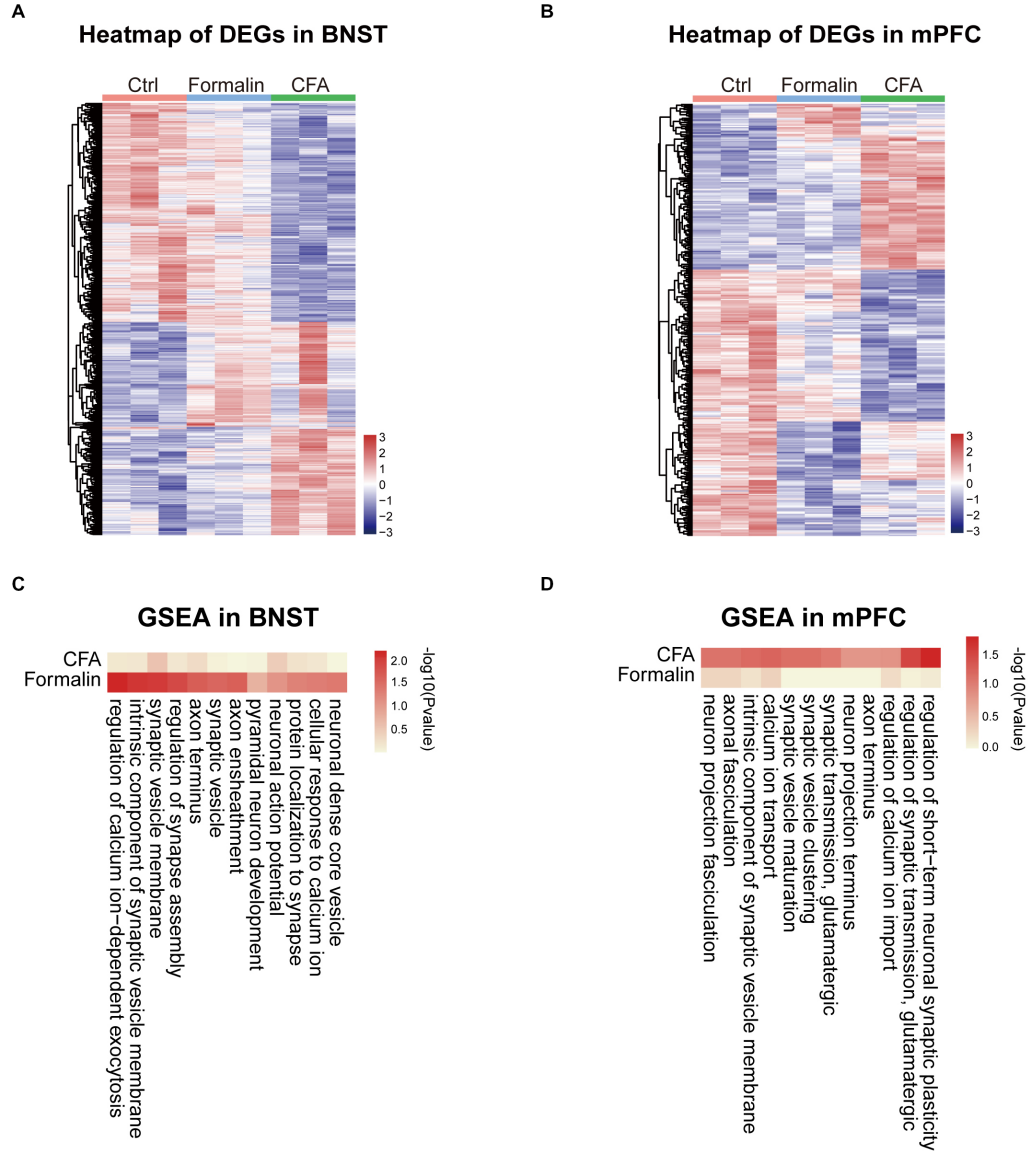
Neuronal Function Related Gene Set Enrichment Analysis (GSEA) of the Two Pain Models in BNST and Prelimbic mPFC. **(A)** Heatmap comparing DEGs expression in the BNST among formalin-, CFA-, and sham-treated mice. Colors indicate the relative expression of DEGs by z-score (*n* = 3 mice each group). **(B)** Heatmap comparing DEGs expression in the prelimbic mPFC among formalin-, CFA-, and sham-treated mice. Colors indicate the relative expression of DEGs by z-score (*n* = 3 mice each group). **(C)** Heatmap comparing GSEA results in the BNST among formalin-, CFA-, and sham-treated mice. Colors indicate -log10 (*p* value). **(D)** Heatmap comparing GSEA results in the prelimbic mPFC among formalin-, CFA-, and sham-treated mice. Colors indicate -log10 (*p* value).

Next, we investigated the functions of the differentially expressed genes (DEGs) in the BNST and prelimbic mPFC. Heatmaps of gene expression showed that formalin and CFA induced substantial amounts of DEGs in both BNST and mPFC (Figures 6A,B). To investigate the function of these DEGs, we performed GSEA on the DEGs. Given the importance of neuronal function and activity in pain- and anxiety-related behaviors, we specifically focused on the Gene Ontology (GO) terms relevant to neuronal excitability and functions, such as synaptic transmission, ion channels, and neural projection. Consistent with higher activation and correlation of the BNST in the formalin-induced acute pain model, the changes in the expression of genes related to neuronal excitability and functions, including neuronal action potential, pyramidal neuron development, and synaptic vesicles, were more significant in the BNST after formalin treatment, compared to CFA treatment (Figure 6C). On the contrary, the changes in the expression of genes, such as glutamatergic synaptic transmission, neuro projection terminus, and calcium ion transport, were more significantly after CFA treatment, compared to those after formalin treatment (Figure 6D). These results support our above findings that the differential activation of the BNST and prelimbic mPFC is associated with differential gene expression, and suggest that the DEGs relevant to neuronal functions might underline the differential activation of the BNST and mPFC and pain-related anxiety-like behaviors induced by acute or persistent pain.

**Figure 7:**
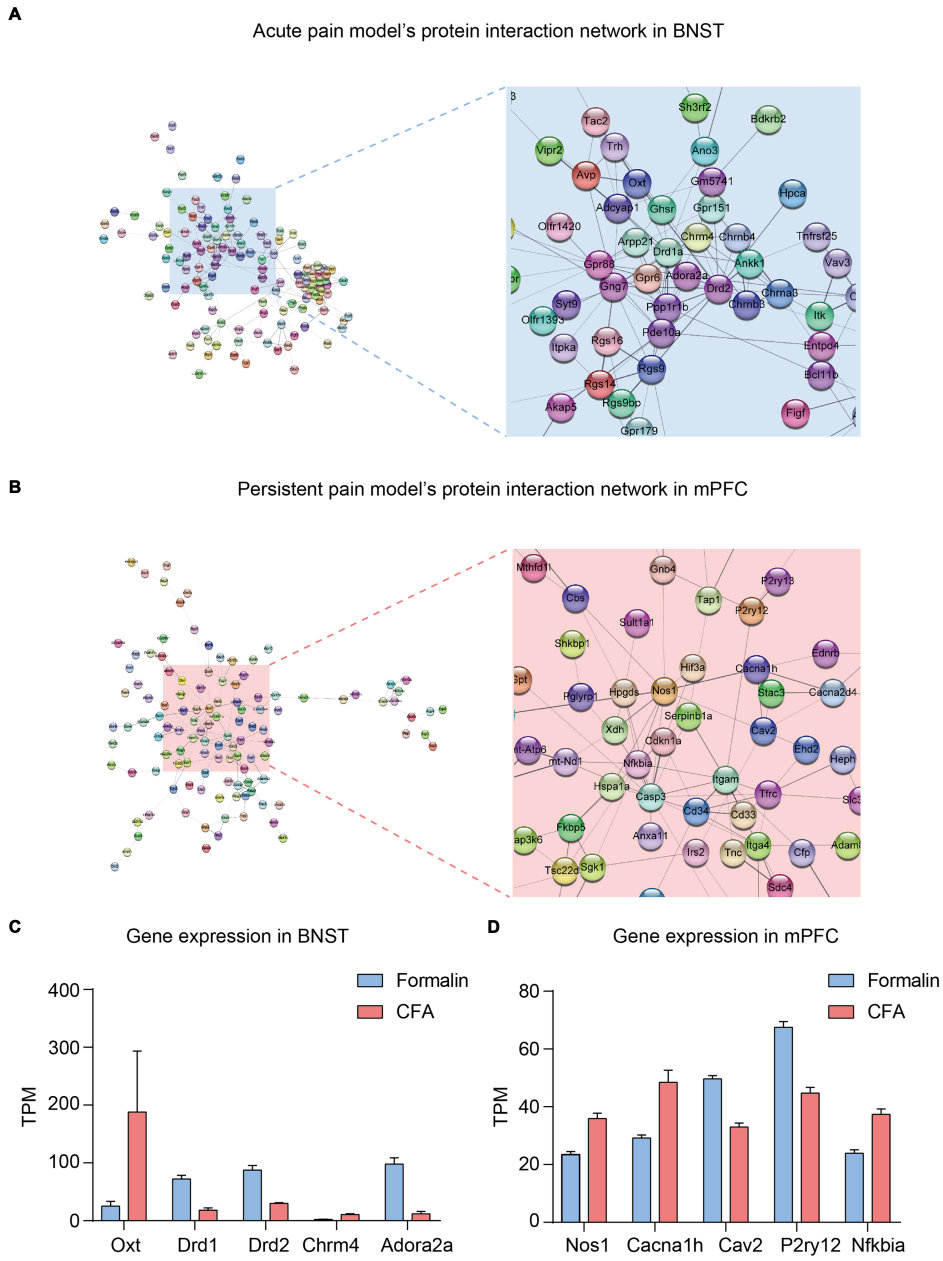
Protein–protein Interaction Network and Key Genes in the BNST and Prelimbic mPFC. **(A)** Protein–protein interaction network of acute pain model in the BNST. **(B)** Protein–protein interaction network of persistent pain model in the mPFC. **(C)** Representative genes and their Transcripts Per Million (TPM) in the BNST under CFA (red) and Formalin (blue) treatment. **(D)** Representative genes and their TPM in the mPFC under CFA (red) and Formalin (blue) treatment.

Finally, we further analyzed the protein–protein interaction network of the DEGs in the BNST and prelimbic mPFC. From the network of the top 300 DEGs in the BNST between the formalin and CFA treatment (Figure 7A), a group of genes related to neuronal functions were clustered together. Notably, Oxt, Drd1, Drd2, Chrna3, and Adora2a lied in the center of the cluster, and their expression levels significantly differed between formalin and CFA treatment (Figures 7A,C). In the network of the mPFC (Figure 7B), Nos1, Cacna1h, Cav2, P2ry12, and Nfkbia lied in the center and exhibited significantly different expression levels between formalin and CFA treatment (Figure 7D). These results further support that neuronal function-related genes might be responsible for the differential activation of BNST and mPFC and pain-related anxiety-like behaviors in acute and persistent pain.

## 4. Discussion

In this study, we find that both acute and persistent pain could induce anxiety-like behaviors; however, they engage different brain regions and mechanisms. The neuronal activity of the BNST^EXT^ neurons is responsible for anxiety-like behaviors induced by acute pain but not by persistent pain. In contrast, the neuronal activity of the mPFC^EXT^ neurons is responsible for anxiety-like behaviors induced by persistent pain but not by acute pain. Transcriptomic analysis reveals that acute and persistent pain induces differential gene expression changes in the BNST and mPFC, and that the DEGs relevant to neuronal functions might underline the differential activation of the BNST and mPFC and pain-related anxiety-like behaviors induced by acute or persistent pain.

Pain adversely affects patients’ quality of life and results in emotional disorders such as anxiety and depression (D'mello and Dickenson, 2008; Baliki and Apkarian, 2015; Yam et al., 2018; Jaromi et al., 2021). Clinically, there are two types of pain, acute and persistent pain (Ren and Dubner, 1999). It has been well established that persistent pain is strongly linked with negative emotions (Baliki et al., 2006; Kohrt et al., 2018; Cohen et al., 2021); however, few studies focused on the impacts and mechanisms of anxiety and depression induced by acute pain. In our study, formalin induces acute pain in the paw of mice, which subsides after about 2 h. Similar to persistent pain, acute pain could also induce anxiety-like behaviors in mice, manifested by the shorter center exploration time in the OF test and the shorter exploration time in the open arms in the EPM test.

The BNST is known to be related to pathological and adaptive anxiety based on neuroimaging and behavioral studies (Straube et al., 2007; Duvarci et al., 2009; Walker et al., 2009; Yassa et al., 2012). The BNST consists of a large number of glutamatergic and GABAergic neurons, which play opposite roles in anxiety behaviors (Poulin et al., 2009; Ch'ng et al., 2018; Kim and Kim, 2021). For example, mice exhibited anxiety-like behaviors when excitatory neurons in the BNST were optogenetically activated. In contrast, the anxiety level was reduced when the inhibitory neurons in the BNST were stimulated (Jennings et al., 2013; Kim et al., 2013). However, whether and how the BNST is involved in pain-related anxiety remains unknown. Utilizing c-Fos expression and RNA sequencing, we first revealed the critical role of the BNST^EXT^ in acute pain-induced anxiety-like behaviors. Importantly, suppressing the neuronal activity of BNST^EXT^ neurons significantly reduced anxiety behaviors induced by acute pain but not persistent pain.

The mPFC is known to be involved in anxiety-related behaviors (Vialou et al., 2014; He et al., 2019; Liu et al., 2020; Li et al., 2021). Previous studies with functional magnetic resonance imaging showed that the mPFC of patients with anxiety is hypoactive (Campbell-Sills et al., 2011; Pfurtscheller et al., 2021). In rodent experiments, bilateral ischemic lesions or activated manipulation of the mPFC could induce anxiety-like behaviors (Parfitt et al., 2017; Deziel and Tasker, 2018). The previous study showed that the intrinsic excitability of mPFC^EXT^ neurons was reduced in persistent pain rats and that the activation of mPFC^EXT^ neurons induced analgesic and anxiolytic effects in mice subjected to persistent pain. In contrast, inhibition of mPFC^EXT^ neurons was anxiogenic in naive mice (Wang et al., 2015). Our study found that mPFC was activated in the persistent pain model and that inhibition of mPFC^EXT^ neurons reduced anxiety-like behaviors in mice. The differences in the pain induction protocol, animal type, and examination time might underline the discrepancy between our study and previous research. These also indicate that the role of mPFC in pain-related anxiety-like behaviors is highly dependent on the type, dynamics, and duration of pain.

The molecular underpinnings of pain-related anxiety have not yet been clearly elucidated. Our RNA-seq analysis indicates that acute and persistent pain induced differential gene expression patterns in the BNST and mPFC. Consistent with the behavioral and c-Fos results, the BNST expressed a higher proportion of upregulated DEGs after acute pain. In contrast, the mPFC showed a higher proportion of upregulated DEGs after persistent pain. These upregulated DEGs might be responsible for the increased neuronal activity in the BNST and mPFC in acute and persistent pain, respectively. Furthermore, enrichment of GO terms relevant to neuronal functions, including glutamatergic synaptic transmission, neuron projection terminus, calcium ion transport, neuronal action potential, pyramidal neuron development, and synaptic vesicles, was found to be different in the two types of pain models. Among them, the BNST showed more significant enrichment in the acute pain model than in the persistent pain model, whereas the mPFC showed opposite patterns. The protein–protein interaction network of either BNST or mPFC showed a cluster of proteins with neuronal function. The Drd1 and Drd2, the D1-like and D2-like dopamine receptors discovered in the BNST network have been reported to have the ability to induce acute pain (Khaleghzadeh-Ahangar et al., 2021). In the mPFC network, inhibitors of Ca_V_2 have long been used to treat persistent pain (Winquist et al., 2005). The importance of Nos1 in persistent pain has also been highlighted (Leanez et al., 2009; Negrete et al., 2011). These results imply the critical roles of these essential genes in the network. Their functions in pain-related anxiety-like behaviors need to be further investigated.

It is important to acknowledge several limitations in the study. Firstly, based on our c-fos staining results, this study focused on the prelimbic region of mPFC. The roles of other regions, including the infralimbic mPFC and anterior cingulate cortex (ACC), in pain-related anxiety-like behaviors need to be further investigated, as their roles in pain are different (Vogt, 2005; Ji and Neugebauer, 2011, 2014; Kiritoshi and Neugebauer, 2015; Koga et al., 2015; Chen et al., 2018; Ong et al., 2019; Kummer et al., 2020; Gao et al., 2023). Secondly, this study only inhibited the activities of excitatory neurons in the mPFC and BNST. Previous studies showed that GABAergic neurons in these regions were also involved in pain and related anxiety-like behaviors (Sabihi et al., 2017; Yamauchi et al., 2022). Therefore, the distinct roles of different cell types in pain-associated anxiety wait to be elucidated. Lastly, the data of this study were collected from male mice. Whether the findings are applicable to females needs further investigation because sex differences in pain perception and anxiety were evident in previous studies (Frot et al., 2004; Rovner et al., 2017; Vambheim and Oien, 2017; Shen et al., 2023).

## 5. Conclusion

Taken together, our results demonstrate that anxiety-related behavior induced by acute and persistent pain are regulated by different brain regions and have distinct transcriptomic patterns, thereby providing new mechanistic insights into anxiety disorders related to different types of pain.

## Data availability statement

The datasets presented in this study can be found in online repositories. The names of the repository/repositories and accession number(s) can be found at: www.ncbi.nlm.nih.gov/geo/, GSE227946.

## Ethics statement

The animal study was reviewed and approved by the Institutional Animal Care and Use Committee of Sun Yat-sen University.

## Author contributions

LH, BL, and SF designed the research studies. SF, SY, JZ, ZL, WL, and XZ performed imaging and behavioral experiment. HZ performed biochemical assays for RNA-seq. SF, YQ, ZL, and XZ analyzed data. SF, YQ, SW, BL, and LH wrote the manuscript. All authors contributed to the article and approved the submitted version.

## Funding

This work was supported by research grants from the National Key R&D Program of China (Grants 2021YFF1200700 to LH), National Natural Science Foundation of China (Grants 82071241, 81871048 to LH and 32071040 to BL), Guangdong Provincial Key R&D Programs (Grant Development of New Tools for Diagnosis and Treatment of Autism 2018B030335001 and Key Technologies for Treatment of Brain Disorders 2018B030332001 to BL and LH), Guangdong Basic and Applied Basic Research Foundation (2023B1515040019 to BL), Guangdong Project (2017GC010590 to BL).

## Conflict of interest

The authors declare that the research was conducted in the absence of any commercial or financial relationships that could be construed as a potential conflict of interest.

## Publisher’s note

All claims expressed in this article are solely those of the authors and do not necessarily represent those of their affiliated organizations, or those of the publisher, the editors and the reviewers. Any product that may be evaluated in this article, or claim that may be made by its manufacturer, is not guaranteed or endorsed by the publisher.
